# Unveil of the role of fungal taxa in iron(III) reduction in paddy soil

**DOI:** 10.3389/fmicb.2023.1334051

**Published:** 2024-01-24

**Authors:** Ming-Jun Li, Xiao-Xin Ye, Yan-Mei Da, Qing-Ye Sun, Guo-Wei Zhou

**Affiliations:** School of Resources and Environmental Engineering, Anhui University, Hefei, China

**Keywords:** fungal taxa, iron(III) reduction, organic metabolism, fermentative iron(III) reducers, respiratory iron(III) reducers

## Abstract

Hitherto, research on iron(III)-reduction has mainly focused on bacteria rather than fungal communities. To acquire insight into fungi involved in iron(III) reduction, typical organic matters (containing cellulose, glucose, lactate, and acetate) and ferrihydrite were used as electron donors and acceptors, respectively, in the presence of antibiotics. After antibiotic addition, microbial iron(III) reduction was still detected at quite high rates. In comparison, rates of iron(III) reduction were significantly lower in cellulose-amended groups than those with glucose, lactate, and acetate under the antibiotic-added condition. Patterns of intermediate (e.g., acetate, pyruvate, glucose) turnover were markedly different between treatments with and without antibiotics during organic degradation. A total of 20 genera of potential respiratory and fermentative iron(III)-reducing fungi were discovered based on ITS sequencing and genome annotation. This study provided an insight into the diversity of iron(III)-reducing fungi, indicating the underestimated contribution of fungi to iron and the coupled carbon biogeochemical cycling in environments.

## Graphical abstract

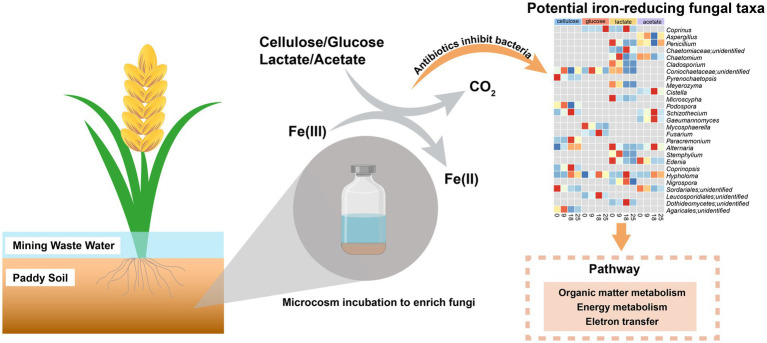

## 1 Introduction

Microbial iron(III) reduction can be catalyzed by dissimilatory iron(III)-reducing microorganisms that rely on the utilization of organic matters (e.g., cellulose, glucose, short-chain fatty acids) as substances to support their growth (Lovley, 1991; Kappler et al., 2021). Iron(III) reducers widely spread under anoxic environments, especially in iron(III)-rich paddy soils (Lovley, 2006; Kappler et al., 2021). Hitherto, almost all explorations on iron(III) reduction focus on bacterial Fe(III) reducers, which are represented by the well-known *Geobacter* spp. and *Shewanella* spp. (Lovley and Phillips, 1988; Myerst and Nealson, 1990). The identified iron(III)-reducing bacteria are phylogenetically diverse, including members belonging to Proteobacteria, Chlorobia, Deferribacteracea, Thermodesulfobacteriaceae, etc. (Weber et al., 2006). In fact, although the abundances of fungal members are lower than those of bacteria, they (yeast, yeast-like, and filamentous species) are widely distributed in terrestrial ecosystems (Blackwell, 2011; Tedersoo et al., 2014). Up to now, iron(III) reduction has only been verified for pure culture of a few species, e.g., *Actinomucor repens*, *Alternaria tenuis*, *Fusarium oxysporum*, *F. solani,* and *Cladosporium* spp. (Ottow and von Klopotek, 1969; Natarajan et al., 1997). Therefore, little is known about the role of iron(III) reduction in fungal communities and the diversity of Fe(III)-reducing fungal taxa in paddy soils.

Organic matters in paddy soils are abundant and complex, encompassing various components such as lignin, cellulose, humic acid, as well as simpler compounds like glucose, lactate, and acetate (Maie et al., 2002; Kögel-Knabner et al., 2010; Yan et al., 2013). Bacteria-mediated Fe(III) reduction is coupled with oxidation of a variety of organic matter, containing fermentative sugars, peptone, organic acids, and H_2_ (Weber et al., 2006; Kappler et al., 2021). Various bacteria which grow through fermentative metabolism are known to transfer just a few electrons to iron(III) oxides (Lovley, 1987; Lovley and Phillips, 1988). Dissimilarly, the Fe(III)-respiring microorganisms are phylogenetically and morphologically diverse and can conserve energy to support growth during iron(III) reduction coupled to oxidation of organic compounds or hydrogen (Lovley, 1987; Lovley and Phillips, 1988; Lovley, 2006). Actually, abundant anaerobic fungi are also involved in cellulose degradation, sugar fermentation, and acetate dissimilation (Hess et al., 2020). Moreover, many of them are recognized for their ability to reduce nitrate or nitrite associated with oxidizing glucose, glycerol, ethanol, acetate and formate (Zhou et al., 2002; Kuwazaki et al., 2003; Aldossari and Ishii, 2021). Certain fungal species, such as *Fusarium oxysporum*, have demonstrated the capability to oxidize acetate coupled with sulfur reduction (Abe et al., 2007). However, limited information is available regarding the response of anaerobic fungal communities to the reduction of iron(III) minerals associated with the oxidation of various organic substances.

We hypothesized that the capacity of anaerobic fungi to reduce iron(III) oxides is linked to the degradation of organic compounds. In this study, we employed anaerobic microcosm cultures using typical paddy soil. We amended the soil with common organics including cellulose, glucose, lactate, and acetate. In order to further highlight the potential role of fungi in iron(III) reduction, treatments with antibiotic combinations were set up simultaneously. The combination of antibiotics aimed to suppress bacterial activity by inhibiting the synthesis of cell wall and ribosomes by vancomycin and streptomycin during the incubation, respectively (Cox et al., 1964; Sieradzki and Tomasz, 1997). The main objective of this study was to: (1) prove that iron(III) reduction occurs in fungi by adding antibiotics to inhibit bacterial activity; (2) identify associated fungal taxa that can potentially reduce iron(III); (3) explore interaction between iron(III)-reducing fungi in response to different organic substrates in a paddy soil.

## 2 Materials and methods

### 2.1 Soil sample collection

Soil samples were collected on 11th in April (2022) from a paddy field irrigated by mining waste water in Yi’an District, Tongling City, Anhui Province, China (117°79′ E, 30°95′ N), currently under soil remediation. The field is planted with rice crop twice a year with no additional fertilization. The temperature at the time of sampling was 14.8°C, and the rice seedlings were not transplanted after harvest. Paddy soil with 10 cm below the water surface, was sampled in three parallels with a sterilized shovel. After removing stones and roots, soil was thoroughly mixed and quickly placed into a large self-sealing bag. The evenly mixed paddy soil was then transported to the laboratory while kept in ice and stored at −20°C before microcosm incubations. pH of this soil was 6.59, which was determined using PHS-3E (Leici, China) according to the method described before (Zhou et al., 2016). The measurement of total organic carbon (TOC) and iron(II) is based on colorimetric assay (Zhou et al., 2016). The total organic carbon and iron in the soil were 30.10 mg/kg and 59.07 g kg^−1^, respectively.

### 2.2 Microcosm incubations

The operation of microcosm incubations was described earlier (Zhou et al., 2016). In brief, the slurry was prepared by mixing paddy soil and sterilized ultrapure water (w: v = 1: 5) in the 2 L beaker in an ultraclean bench. After homogenization, the slurry was exposed to N_2_/CO_2_ (v/v = 80/20) for 1 h, and aliquots (40 mL) of the soil slurry were transferred into 50 mL serum bottles. The headspace of the medium was flushed with N_2_/CO_2_ for 20 min and then vials were capped with butyl stoppers and aluminum seal. Pre-incubation of the slurry was performed at 30°C in the dark for 96 h, aiming to consume the soluble organic matter including sugars and short-chain organic acids originally present in the paddy soil. In order to investigate the role of fungi in iron(III) reduction in the paddy soil, a total of eight ferrihydrite-added treatments (*n* = 3, each) were established using various organic matters as electron donors: (1) the slurry was amended with 4 g/L carboxymethylcellulose (termed as cellulose); (2) the slurry was amended with 4 g/L carboxymethylcellulose and antibiotics containing 50 mg/L streptomycin and 50 mg/L vancomycin (cellulose + antibiotics); (3) the slurry was amended with 1 mM glucose (glucose); (4) slurry was amended with 1 mM glucose and antibiotics containing 50 mg/L streptomycin and 50 mg/L vancomycin (glucose + antibiotics); (5) the slurry was amended with 2 mM sodium lactate (lactate); (6) the slurry was amended with 2 mM sodium lactate and antibiotics containing 50 mg/L streptomycin and 50 mg/L vancomycin (lactate + antibiotics); (7) 3 mM sodium acetate (acetate); (8) the slurry was amended with 3 mM sodium acetate and antibiotics containing 50 mg/L streptomycin and 50 mg/L vancomycin (acetate + antibiotics). Ferrihydrite was prepared by adding KOH solution to Fe(NO_3_)_3_·9H_2_O solution and then making a pH adjustment to 6.8–7.2 according to the previous description (Zhou et al., 2016).

### 2.3 Dynamic sampling and chemical analyses

The serum bottles were dynamically sampled on days 0, 9, 18, and 25 during incubation with sterile syringes in an ultra-clean bench. Then, 40 mM sulfamic acid was used for iron(II) extraction, and total Fe was extracted with sulfamic acid (80 mM) and hydroxylamine hydrochloride (80 mM) (v:v = 1:1) (Zhou et al., 2016). After centrifugation of remaining culture suspensions at 10,000 g for 10 min, sediments were used for DNA extraction based on the kit manufacturer’s instructions (MP Biochemicals; Solon, OH, United States), while supernatants were used to measure glucose according to the anthrone method (Updegraff, 1969) and organic acids including acetate, lactate and pyruvate using ion chromatograph (Dionex ICS-1500 system; Diones, Sunnyvales, CA).

### 2.4 High-throughput sequencing and data analysis

Fungal amplification of the ITS2 region was performed using primers Gis7 (GTGARTCATCGARTCTTTG) and ITS4 (TCCTCCGCTTATTGATATGC) (Geng et al., 2022). PCR conditions were pre-denaturated at 94°C for 3 min, then 35 cycles were performed at 94°C for 30 s, 58.3°C for 30 s, and 72°C for 45 s, and finally extended at 72°C for 10 min. The PCR products were then purified, quantified, pooled, and sequenced on an Illumina Miseq PE 250 Platform (Novogene, Beijing, China). Sequencing data were analyzed using QIIME2 (version 2021.11) following the online instructions (Bolyen et al., 2019). Deblur algorithm was used to determine amplicon sequence variants (ASVs) for the sequencing data by denoising in the QIIME2 platform, and UNITE v2020.2 reference databases were used to annotate fungal taxonomy (Bolyen et al., 2019). R software loaded with EdgeR package was utilized for statistical analyses, and fungal species with a significant increase (*p* < 0.05) in abundance between days were denoted as active taxa during the incubation. The ITS2 gene sequences have been deposited in the NCBI database with accession number PRJNA887227.

*16S rRNA* gene of bacteria on day 9 during incubations was quantified by fluorogenic quantitative PCR (qPCR), and the condition and primer has been described before (Zhou et al., 2016). In detail, The V4-V5 region of the *16S rRNA* gene was amplified using primers 515F (GTGCCAGCMGCCGCGG) and 907R (CCGTCAATTCMTTTRAGTTT). qPCR conditions were predenaturated at 94°C for 3 min, then 35 cycles were performed at 94°C for 30 s, 55°C for 30 s, and 72°C for 45 s, and finally extended at 72°C for 10 min.

### 2.5 Metagenomic assembly, genome binning, gene annotation and its gene alignment

A total of 46 assembled fungal MAGs (Supplementary Table S1), which are phylogenetically close to the active fungal taxa identified during incubation, were retrieved from the NCBI database (Supplementary Tables S2–S4). The MAGs were sequenced using several methods, including Illumina HiSeq, Illumina MiSeq, PacBio, and Nanopore, and the information about the sequencing method, assembly method, and the relevant references are detailed in Supplementary Table S1. Protein-coding regions were predicted using Prodigal (version 2.6.3) with the “-p meta” option (Hyatt et al., 2010). The eggNOG-mapper (Huerta-Cepas et al., 2017), KEGG server (BlastKOALA) (Kanehisa et al., 2016), InterProScan tool (5.44–79.0) (Jones et al., 2014), and Diamond (0.9.22)-connected NCBI-nr database searched in May 2022 (*E*-value cutoff ≤1e-5) were employed to annotate the protein-coding regions.

Fungal ITS2 gene sequences, including ITS2 gene from Illumina sequencing and extracted from fungal MAGs obtained from NCBI database, were aligned using NCBI BLAST.^1^ Ribosomal ITS2 genes in fungal MAGs were extracted by ITSx (version 1.1.3^2^).

## 3 Results

### 3.1 Iron(III) reduction in treatments with or without antibiotics

**Figure 1:**
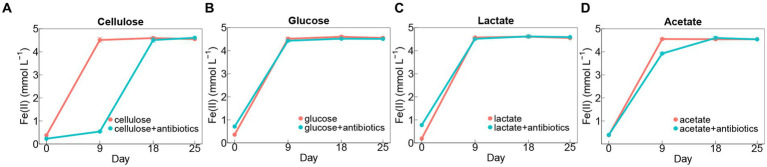
Dynamics of Fe(II) concentrations in treatments amended with cellulose **(A)**, glucose **(B)**, lactate **(C)**, and acetate **(D)** during days of incubation with or without antibiotics. Error bars represent standard deviations of three replications.

For almost all the groups, it displayed rapid iron(III) reduction in the first 9 days during incubation (Figures 1A–D). The rates of iron(III) reduction were around 0.46–0.49 mmol L^−1^ day^−1^ in no-antibiotics treatments with glucose, lactate, and acetate during 9-days incubation (Figures 1B–D), which were significantly (*p* < 0.05) higher than those in cellulose treatments without antibiotics (Figure 1A). The addition of antibiotics markedly decreased iron(III)-reducing rates in cellulose treatments during incubations (Figure 1A). Whereas, no significant difference in rates of iron(III) reduction was found between other setups (glucose, lactate, and acetate) with and without antibiotic addition (Figures 1B–D).

### 3.2 Decomposition of organic matters in treatments with or without antibiotics

**Figure 2:**
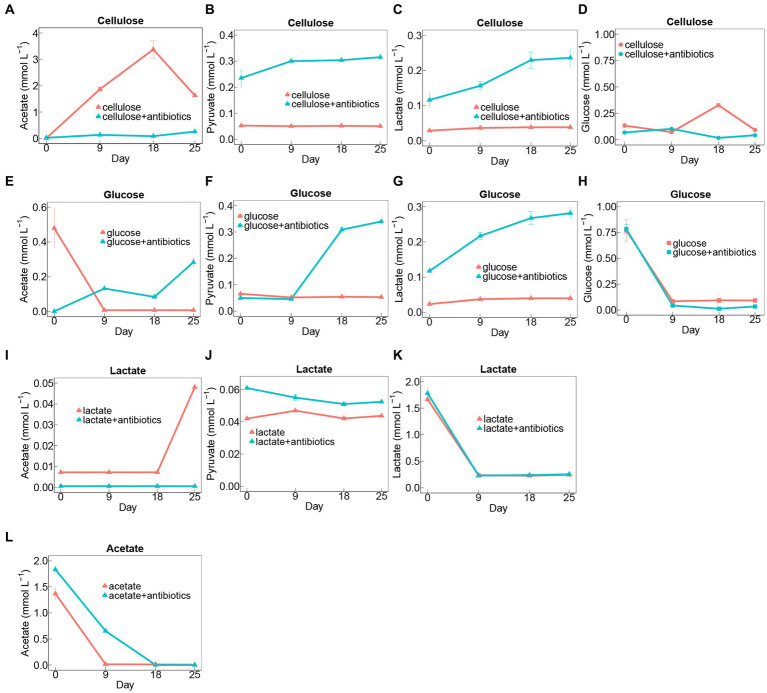
Time course of metabolite concentrations in treatments amended with cellulose **(A–D)**, glucose **(E–H)**, lactate **(I–K)**, and acetate **(A)** during days of incubation with or without antibiotics. Error bars represent standard deviations of three replications.

Cellulose degradation was accompanied by the generation of intermediates, including glucose, lactate, pyruvate, and acetate (Figures 2A–D). Notably, there were fluctuations in the concentrations of glucose and acetate, reaching peak levels of 0.33 and 3.38 mmol L^−1^ on day 18, respectively. Conversely, the concentrations of pyruvate and lactate showed no significant changes in cellulose treatments without antibiotics during the 25-day incubation period (Figures 2A–D). In contrast, in setups with antibiotics added to cellulose, there was a conspicuous accumulation of pyruvate and lactate (Figures 2B,C). However, lower concentrations of acetate and glucose accumulated in cellulose amendments with antibiotics than those without antibiotics (Figures 2A,D).

Generation of lactate, pyruvate, and acetate was detected in glucose-amended groups during 25-day incubation (Figures 2E–H). Glucose was exhausted on day 9 during the incubation, and there was no apparent distinction in rates and extents of glucose degradation between treatments with antibiotics and without antibiotics (Figure 2H). Additionally, the intermediates including lactate, pyruvate, and acetate evidently increased in glucose-adding groups with antibiotics, whereas acetate rapidly accumulated at the beginning of the incubation and then sharply depleted on day 9 in glucose treatments amended without antibiotics (Figures 2E–G).

Similarly, the addition of antibiotics had no significant effect on the rate of lactate degradation (Figure 2K). Moreover, it was found higher amounts of pyruvate were generated in the antibiotic-present setups than in the antibiotic-absent ones, nevertheless, which exhibited an opposite trend to the acetate accumulation for these two treatments with lactate during 25-day incubation (Figures 2I,J).

With regard to treatments with acetate, additional antibiotics substantially inhibited its degradation rates during incubation (Figure 2L).

### 3.3 Active fungal taxa in treatments with organic matters amended with and without antibiotics

**Figure 3:**
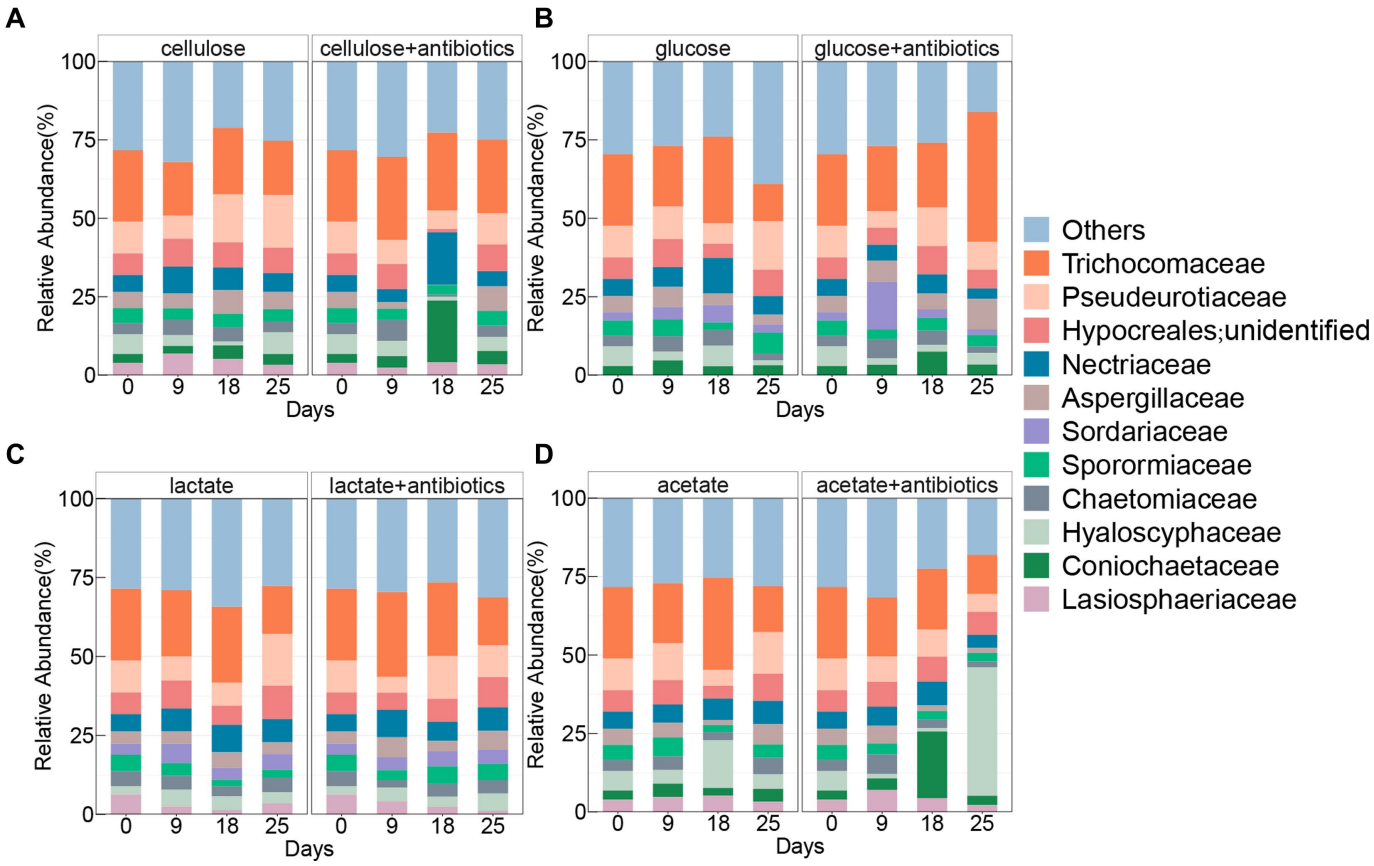
Pattern of fungal community of top 10 taxa at the family level in treatments amended with cellulose **(A)**, glucose **(B)**, lactate **(C)**, and acetate **(D)** during days of incubation with or without antibiotics.

The family Trichocomaceae was found with the highest relative abundances in all treatments with and without antibiotics (Figure 3), which shared 7 other families containing Pseudeurotiaceae, unclassified Hypocreales, Nectriaceae, Chaetomiaceae, Aspergillaceae, Sporormiaceae, Chaetomiaceae, and Hyaloscyphaceae enriched during the incubation with and without antibiotics (Figure 3). Comparably, there was no enrichment of Lasiosphaeriaceae and Coniochaetaceae in antibiotic-present/absent setups with glucose and lactate, respectively (Figure 3). In addition, the family Sordariaceae was specifically detected in treatments with glucose and lactate under conditions with and without antibiotics (Figure 3).

**Figure 4:**
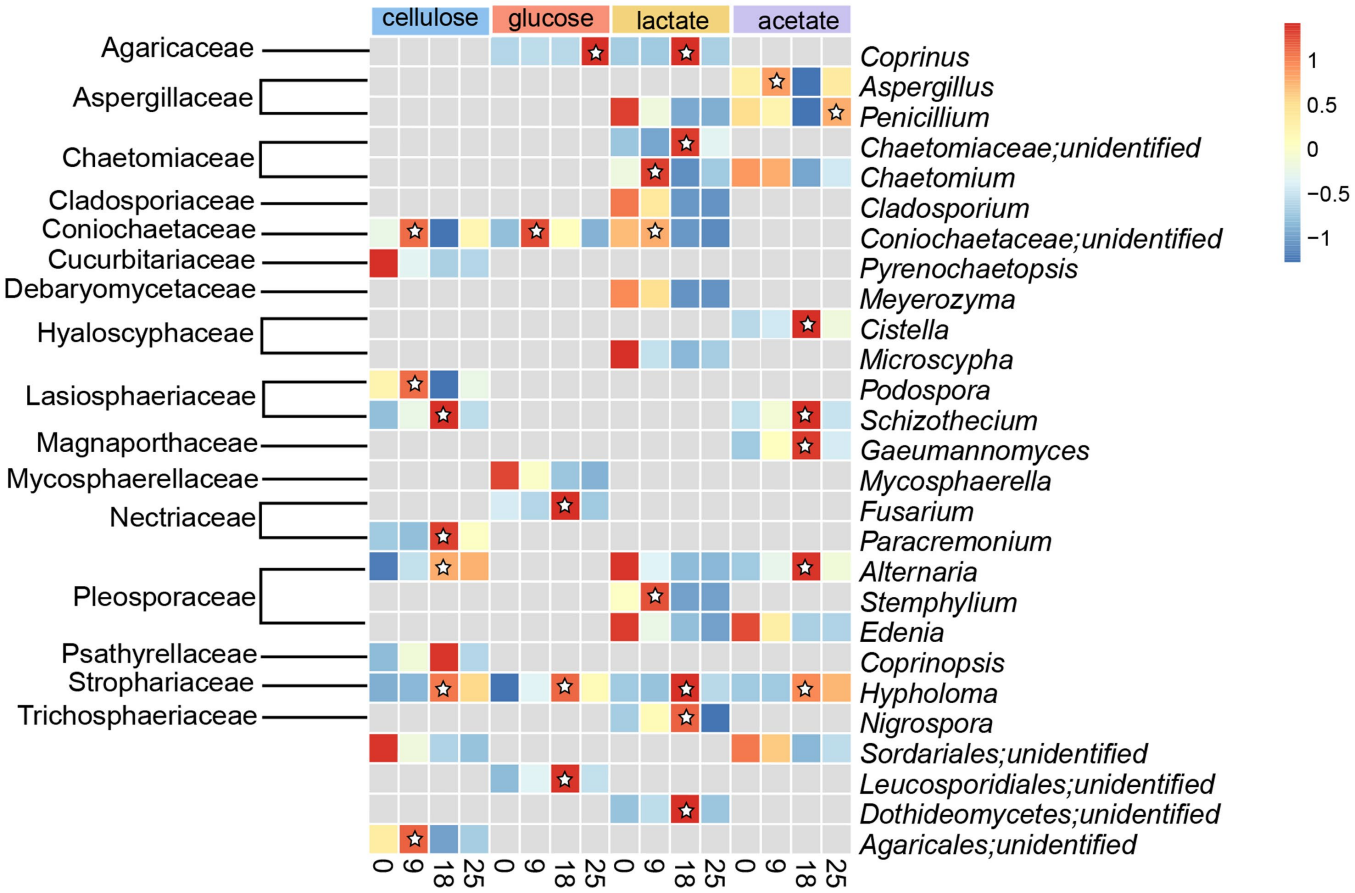
Heatmap of relative abundances of fungal taxa with significant difference (*p* < 0.05) in the genus level detected in treatments amended with cellulose, glucose, lactate and acetate during days of incubation without antibiotics. ☆represented abundances of taxa increased during the incubation, which were denote as active taxa in this study.

Active fungal taxa in this study referred to the genera of fungal whose abundances significantly increased (*p* < 0.05) during incubations with cellulose, glucose, lactate, and acetate. A total of 10, 6, 14, and 10 active genera were detected during incubation with cellulose, glucose, lactate, and acetate under no addition of antibiotics, respectively (Figure 4). Moreover, all active genera found in organic matter degradation were members belonging to the top 10 most abundant families (Figures 3, 4). The relative abundances of 7 genera including unidentified Coinochaetaceae, *Podospora*, *Schizothecium*, *Paracremonium*, *Alternaria*, *Coprinopsis*, *Hypholoma*, and unidentified *Agricales* increased, especially, in the first 9 or 18 days, whereas, it was observed with a downward tendency in relative abundances of unidentified Pyrenochaetopsis and unidentified Sordariales during the incubation with cellulose (Figure 4). There were 5 (containing *Coprinus*, unidentified Coinochaetaceae, *Fusarium*, *Hypholoma,* and unidentified Leucosporidiales) and 1 (*Mycosphaerella*) genera with increase and decrease in relative abundances in no antibiotic-amended glucose treatments, respectively (Figure 4). In addition, the number of genera whose abundances increased with lactate and acetate addition were 7 and 6 during incubation for the first 9 or 18 days of incubation, while the ones with decreasing abundances were more diverse in treatments with lactate (7 genera) and acetate (4 genera), respectively (Figure 4).

### 3.4 Physiological capability of active fungal taxa revealed by metagenome analysis

**Figure 5:**
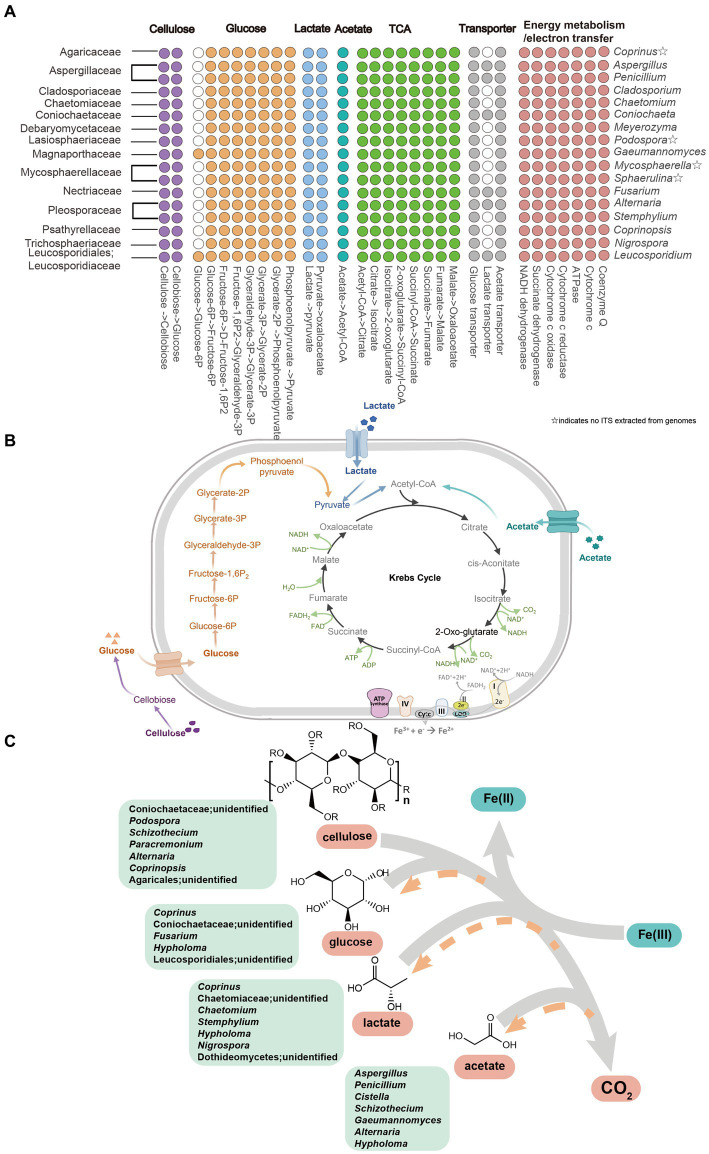
Metabolic pathway of cellulose, glucose, lactate and acetate in active fungal taxa. **(A)** Gene homologs in active fungal MAGs. **(B)** Proposed active pathway in fungal taxa. Pathways were constructed based on fungal MAGs (Supplementary Table S1). ☆indicates that ITS2 sequences were not extracted from the fungal MAGs. **(C)** Iron(III)-reducing potential fungi including fermentative and four organic matters metabolism with the reduction of Fe(III) to Fe(II) in cellulose, glucose, lactate and acetate amended setups. The gray arrows represented that cellulose, glucose, lactate and acetate degraded into CO_2_ by fungal members and the orange arrows showed the products of glucose, lactate and acetate in the process of cellulose decomposition.

To comprehensively elucidate the metabolic capabilities of active fungal taxa, 46 fungal MAGs were selected based on the sequence alignment between the ITS2 gene extracted from MAGs and relevant ASV sequences detected in this study (Supplementary Tables S1–S3), and most of the identity values were higher than 95% (Supplementary Table S4). Metagenomic inference revealed that all of these active fungi harbor genes encoding complete pathways for cellulose degradation, leading to the production of glucose (Figure 5; Supplementary Tables S5–S8). Moreover, all of these MAGs possess genes encoding enzymes responding to glucose fermentation, lactate and acetate utilization, Krebs cycle, energy metabolism, and electron transfer (Figure 5; Supplementary Tables S5–S8). However, MAGs excluding genera *Gaeumannomyces* and *Leucosporidium* lack genes catabolizing glucose to produce glucose-6p (Figure 5; Supplementary Figure S1; Supplementary Tables S5–S8). In addition, genes involved in transporting glucose and acetate are identified in all the MAGs, nevertheless, being absent of genes driving lactate uptake for genera such as *Coprinus*, *Cladosporium*, *Chaetomium*, *Meyerozyma*, etc. (Figure 5; Supplementary Tables S5–S8).

## 4 Discussion

### 4.1 Activity of fungi to reduce iron(III) in a paddy soil

Paddy soil contains a rich supply of organic matter, including cellulose, glucose, lactate, and acetate (Lovley and Phillips, 1986). Additionally, the bacterial community plays a crucial role in the process of iron reduction in paddy soils (Weber et al., 2006; Kappler et al., 2021). The abundances of bacterial *16S rRNA* genes in the treatments with antibiotics were significantly (*p* < 0.0001) decreased compared to incubation without antibiotics (Supplementary Figure S3). However, treatments with organics were still detected with the exhaustion of iron(III) within 9 days after antibiotic addition (Figure 1) suggesting the strong capability of iron(III) reduction by fungal communities in treatments with cellulose, glucose, lactate, and acetate.

During the ferrihydrite reduction, cellulose decomposition occurred with the production of glucose, pyruvate, lactate, and acetate in treatments with and without antibiotics (Figures 2A,D), unveiling the existence of cellulolytic fungi during the incubation. Cellulose hydrolysis, which may be followed by forming cellobiose as intermediates, can be catalyzed by 1,4-beta-D-glucan cellobiohydrolase, 1, 4-beta cellobiohydrolase and beta-glucosidase to produce glucose (Leschine, 1995). Since this process contains no redox reactions, there would be no transfer of electrons for iron(III) reduction. By contrast, the oxidation of glucose and lactate can supply prolific electron donors for dissimilatory iron(III) reducers (Weber et al., 2006; Kappler et al., 2021). Throughout the incubation with antibiotics, the fluctuations in concentrations of glucose, pyruvate, and lactate (Figures 2E–K) indicated fermentative iron(III)-reducing fungal community were active to employ cellulose, glucose, and acetate as electron donors. In addition, the strong ability of respiratory iron(III) reduction by fungal members was revealed by the rapid decrease in acetate and iron(III) (Figures 1, 2I) during the acetate-added groups under conditions with or without antibiotics (Lovley, 2006).

### 4.2 The diversity of fermentative iron(III)-reducing fungi in a paddy soil

Iron-reducing organisms were divided into two categories, respiratory and fermentative iron reducers. Fermentative iron fungi concurrently consume fermentative organic substrates (e.g., glucose or pyruvate) to produce Fe(II) and acquire a small amount of energy from iron reduction to support growth (Dong et al., 2017). The potential fermentative iron(III)-reducing fungal members constituted five active genera for glucose oxidation, including *Coprinus*, unidentified Coniochaetaceae, *Fusarium*, *Hypholoma*, and unidentified Leucosporidiales, and 7 active genera for lactate utilization, e.g., *Coprinus*, *Chaetomium*, *Stemphylium*, *Hypholoma*, *Nigrospora*, etc. For glucose metabolization, unidentified Leucosporidiales (Figures 4, 5A,C), which can refer to the phylogenetically related Leucosporidium, may take the responsibility to catabolize glucose with generating glucose-6-phosphate and other active taxa may jointly complete glycolysis (Figures 4, 5A). MAGs of all the active genera encode enzymes linked to lactate catabolism and the Krebs cycle, and *Coniochaeta*’s genome is equipped with a lactate transporter-relevant gene (Figure 5), which further supports their ability to utilize lactate during incubation. As a result, a total of 10 genera were considered as possible fermentative iron(III)-reducing fungal communities.

### 4.3 The diversity of respiratory iron(III)-reducing fungi in a paddy soil

Conversely, respiratory iron(III)-reducing fungi metabolize non-fermentative substances (eg. acetate) to reduce ferrihydrite. Moreover, as a non-fermentative substrate is used as an electron donor only, the electron transfer strategy determines the occurrence of dissimilatory iron reduction (Nealson and Saffarini, 1994). There were 7 genera (*Aspergillus*, *Penicillium*, *Cistella*, *Schizothecium*, *Gaeumannomyces*, *Alternaria*, and *Hypholoma*) affiliated to 6 families, actively taking up acetate during ferrihydrite reduction (Figures 4, 5A,C), were though as potential respiratory iron(III)-reducing fungal microorganisms attribute to the non-fermentable property of acetate. Moreover, the complete metabolic pathway of acetate equipped by most active taxa-relevant MAGs (Figures 5A,B) is consistent with this conclusion. Up to date, the diversity of fungal species associated with iron(III) reduction was limitedly documented except for members belonging to the genus *Fusarium*, *Alternaria*, and *Aspergillus* (Ottow and von Klopotek, 1969). Both *Fusarium* and *Alternaria* show activity to reduce hematite through using glucose as the substrate (Ottow and von Klopotek, 1969). Our results align with the former, while the latter specifically utilized acetate during incubation in our study (Figure 4). The species *Aspergillus niger* has been reported to bioleach iron from a clay mineral under acidic conditions with pH ranging from 1.7 to 3 (Hosseini et al., 2007). Besides, Lalinská-Voleková et al. have reported that *Coprinus* is abundant in environments rich in hydrous ferric oxides (Lalinska-Volekova et al., 2022). Similarly, *Stemphylium botryosum* (belongs to the genera *Stemphylium*) showed a high capacity to use citrate to reduce external ferric iron (Manulis et al., 1987). *Penicillium* sp. is capable of leaching Fe^3+^ from jarosite minerals and reducing Fe^3+^ to Fe^2+^ (Oggerin et al., 2014). The functional genes involved in iron(III) reduction were not identified yet, leading to difficulty in the identification and confirmation of functional iron(III)-reducing fungal candidates. However, the relevant MAGs of these potential fermentative iron(III)-reducing fungi harbored genes encoding nitrate reductase, hinting at their possible role in nitrate reduction under anaerobic conditions. Most iron-reducing microorganisms (facultative anaerobic bacteria, fungi, and yeasts) are simultaneously nitrate-reductase inducible (Ottow, 1969; Ottow and von Klopotek, 1969; Ottow and Glathe, 1971).

### 4.4 Contribution of potential iron(III)-reducing fungi to cellulose degradation

Most of the active fungal members such as Coniochaetaceae, Lasiosphaeriaceae, Nectriaceae, and Pleosporaceae belonged to the phylum *Ascomycota* (Figure 4), which has been reported as saprophytic fungi (Weber et al., 2011; Qin et al., 2014). Specifically, the relevant MAGs of these active fungal taxa, including unidentified Coniochaetaceae, *Podospora*, *Schizothecium*, *Paracremonium*, *Alternaria*, *Coprinopsis* and unidentified Agaricales, encode enzymes including 1,4-beta-D-glucan cellobiohydrolase, 1, 4-beta cellobiohydrolase, glucan 1,3-beta-glucosidase, glycoside hydrolase and beta-glucosidase to participate in breaking bond of cellulose with yielding glucose (Figures 5A–C; Supplementary Tables S5–S8). This enzymatic profile aligned well with the observed glucose production during cellulose incubation (Figure 2D). Moreover, these 7 active fungal taxa are capable of hydrolyzing cellulose in anaerobic or aerobic environments such as damp chamber cultures, plant biomass, wheat straw-amended soil, mangrove and cattle rumen (Croan, 2000; Liu et al., 2010; Šnajdr et al., 2010; Deep et al., 2014; Poidevin et al., 2014; Carrillo et al., 2016; Velasco-Rodríguez et al., 2022). Intermediates of cellulose containing glucose, lactate, pyruvate, and acetate were identified in the cellulose incubation with antibiotics amendments (Figures 2A–D), reflecting the fungal activity in further breaking down cellulose. With the oxidation of glucose, lactate, pyruvate, and acetate, the active members in cellulose treatments containing unidentified Coniochaetaceae, *Schizothecium,* and *Alternaria* probably (Figures 2, 4) were likely to accept the released electrons to reduce ferrihydrite, which offers a plausible explanation for their increased abundances during the incubation.

## 5 Conclusion

In summary, the introduction of antibiotics had a pronounced effect on the iron(III) reduction rate within cellulose-amended groups, markedly slowing down the process, while exhibiting negligible impact on the other groups. Furthermore, metabolic rates of organic substrates and intermediates significantly altered with antibiotics amendment during incubation. Besides, there were several active genera for the metabolism of cellulose (e.g., *Podospora*, *Schizothecium*, *Paracremonium*, *Alternaria*, *Coprinopsis*), glucose (e.g., *Coprinus*, *Fusarium*, *Hypholoma*), lactate (e.g., *Coprinus*, *Chaetomium*, *Stemphylium*) and acetate (e.g., *Aspergillus*, *Penicillium*, *Cistella*), whose genomes possess complete metabolic pathways for degradation of the typical organic matters. Furthermore, the investigation revealed the presence of 20 genera (e.g., *Fusarium*, *Alternaria*) with the potential for respiratory and fermentative iron(III) reduction particularly in experimental setups amended with four distinct organic substances. In conclusion, it was proven that the potential and diversity of iron(III)-reducing fungi, clarified the underestimated function of fungi in directly driving the carbon and iron biogeochemical cycling, and provided a new perspective for further understanding of carbon and iron cycling in environments.

## Data availability statement

The datasets presented in this study can be found in online repositories. The names of the repository/repositories and accession number(s) can be found in the article/Supplementary material.

## Author contributions

M-JL: Data curation, Formal analysis, Investigation, Methodology, Visualization, Writing – original draft. X-XY: Conceptualization, Investigation, Methodology, Supervision, Visualization, Writing – review & editing. Y-MD: Investigation, Methodology, Software, Visualization, Writing – review & editing. Q-YS: Conceptualization, Formal analysis, Methodology, Supervision, Writing – review & editing. G-WZ: Conceptualization, Funding acquisition, Methodology, Project administration, Supervision, Writing – review & editing.

## Footnotes

^1^ https://blast.ncbi.nlm.nih.gov/Blast.cgi

^2^ https://microbiology.se/tag/itsx/
